# Supplementary material for: The Vibrio vulnificus stressosome is an oxygen-sensor involved in regulating iron metabolism
Source: Commun Biol. 2022 Jun 27;5:622. doi: 10.1038/s42003-022-03548-w (PMC9237108; doi:10.1038/s42003-022-03548-w)
Supplement: Supplementary file 2 — Supplementary Information (new) [file 42003_2022_3548_MOESM2_ESM.pdf]

SUPPLEMENTARY INFORMATION

The *Vibrio vulnificus* stressosome is an oxygen-sensor involved in regulating iron metabolism

Veronika Heinz[1]*, Wenke Jäckel[2]*, Susann Kaltwasser[3]*, Laura Cutugno[4], Patricia Bedrunka[5], Anica Graf[2], Alexander Reder[6], Stephan Michalik[6], Vishnu M. Dhople[6], M. Gregor Madej[1], Maria Conway[5], Marcus Lechner[5], Katharina Riedel[2], Gert Bange[5], Aoife Boyd[4], Uwe Völker[6], Richard J Lewis[7§], Jon Marles-Wright[8], Christine Ziegler[1#] and Jan Pané-Farré[5#]

1) Department of Biophysics II / Structural Biology, University of Regensburg, Regensburg 93053, Germany
2) Department of Microbial Physiology and Molecular Biology, University of Greifswald, Greifswald 17487, Germany
3) Max Planck Institute of Biophysics, Max-von-Laue-Strasse 3, 60438 Frankfurt am Main, Germany
4) Discipline of Microbiology, School of Natural Sciences, Molecular Pathogenesis Research Group, National University of Ireland Galway, Galway, Ireland.
5) Center for synthetic Microbiology (SYNMIKRO) & Department of Chemistry, Philipps-University Marburg, Karl-von-Frisch-Strasse 14, 35043 Marburg
6) Interfaculty Institute for Genetics and Functional Genomics, University Medicine Greifswald, Greifswald, Germany
7) Biosciences Institute, Newcastle University, Newcastle upon Tyne NE2 4HH, UK
8) School of Natural and Environmental Sciences, Newcastle University, Newcastle upon Tyne, NE1 7RU, UK

§) Current address: The Royal Society for the Protection of Birds, The Lodge, Potton Road, Sandy, Bedfordshire SG19 2DL

# Correspondence:

email: jan.panefarre@chemie.uni-marburg.de, telephone: +49-6421 28 22211

email: Christine.ziegler@biologie.uni-regensburg.de telephone: +49-941 943 3030

SUPPLEMENTARY FIGURES

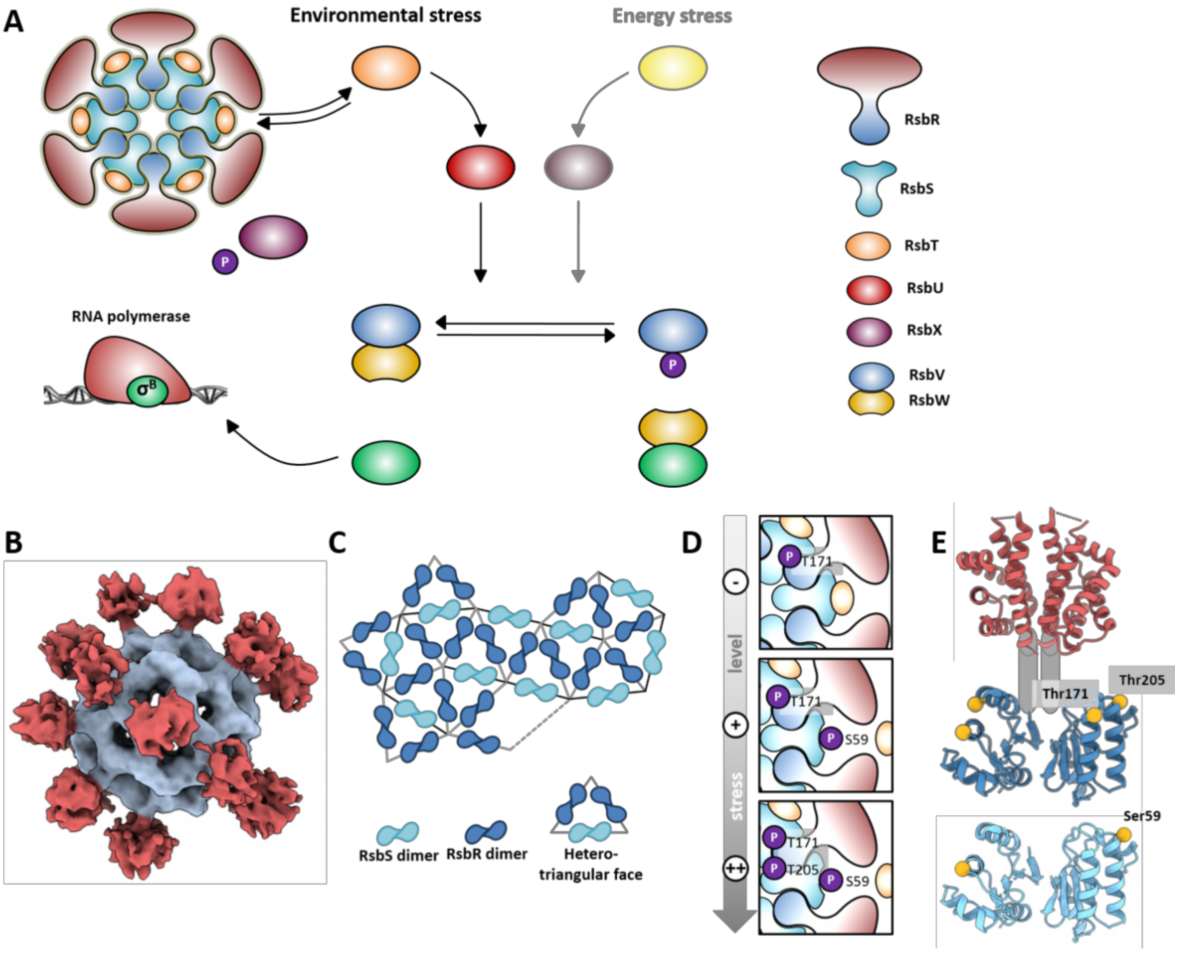

Supplementary figure 1. **The *Bacillus subtilis* stressosome in a nutshell**

a) The SigB regulatory cascade. Environmental stress sensed by the stressosome leads to RsbT release and RsbT-dependent activation of the phosphatase RsbU[1,2]. Activated RsbU dephosphorylates the anti-sigma factor antagonist RsbV, triggering a partner-switching cascade involving the release of the stress sigma-factor, SigB, from its inhibitory complex with RsbW[3–5]. In addition to being an anti-sigma factor, RsbW also has RsbV-specific kinase activity, which leads to dissociation of the RsbW:RsbV complex[6,7]. b) The cryo-EM density map of the *B. subtilis* RsbR:RsbS stressosome (EMD 1555) reveals the molecular architecture of the complex[8]. The stressosome core (grey) is formed by the STAS domains of RsbR and RsbS. The N-terminal sensory domains of RsbR, also termed turrets (red), protrude from the core. While the STAS domain core exhibits icosahedral symmetry features, the arrangement of RsbR in the complex follows D2 point-group symmetry, resulting in an overall pseudo-icosahedral symmetry[8]. c)

The "Unfolded" view of the stressosome complex reveals two bands of five RsbS dimers (cyan) wrapping around the complex including 20 RsbR dimers (blue), resulting in a 2:1 $RsbR_2$:$RsbS_2$ stoichiometry. The smallest repeating unit of the complex thus consists of hetero-triangular faces, comprised two RsbR dimers and one RsbS dimer. Stressosome functionality is controlled by RsbT-dependent phosphorylation at conserved residues in the STAS domains of RsbS and the RsbR paralogs (d)[9–11]. While pre-stress phosphorylation of Thr171 in RsbR is essential to put the stressosome in a sensitive state, phosphorylation at Thr205 desensitizes the stressosome to limit activation under conditions of severe persistent stress[12]. Phosphorylation of Ser59 in RsbS leads to release of RsbT from the stressosome surface[9,11]. Phosphorylations within the stressosome are removed by RsbX to reset the stressosome[13]. Energy limitation is sensed and transmitted by the RsbQ/RsbP pair[14,15]. e) The RsbR dimer is composed of the C-terminal STAS domain (blue, represented by PDB ID 2VY9) and the N-terminal sensory domain (red, represented by PDB ID 2BNL), which are connected by a pair of linker helices (grey)[16]. The position of regulatory phosphorylation sites in the STAS domains of RsbR (Thr171 and Thr205) is shown in gold. The RsbS dimer (cyan, represented by PDB ID 2VY9) also exhibits the STAS domain fold, but comprises only one phosphorylation site (Ser59) per monomer (gold)[8].

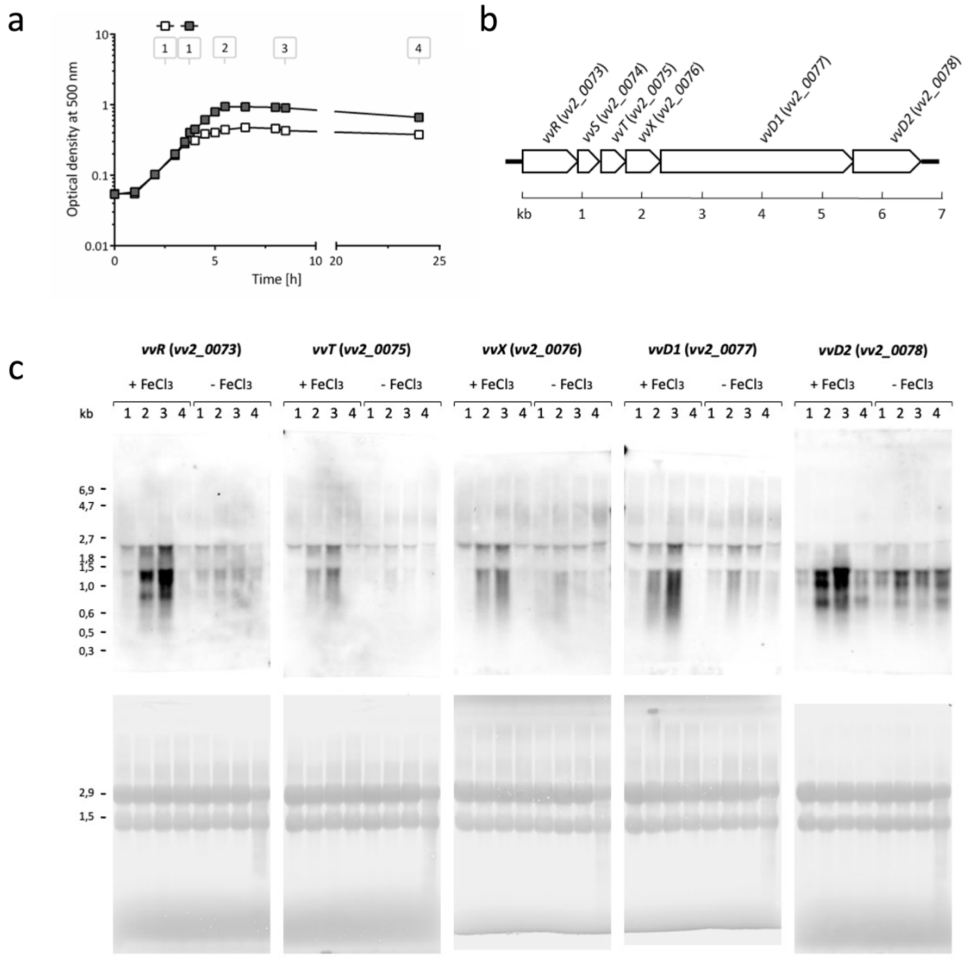

Supplementary figure 2. **Expression of the *V. vulnificus* stressosome gene cluster.** For growth phase and iron-dependent analysis of stressosome gene cluster transcription, cells were harvested during mid-exponential phase, upon entry into stationary phase – triggered by glucose exhaustion, as well as four and 20 hours into the stationary phase. The four harvesting time points are indicated by squares shown above the growth curve. Since the non-iron supplemented culture entered the transient phase at an earlier time point compared to the supplemented culture, the exponential phase sample was harvested earlier (a). The hypothetical organization of the stressosome gene cluster including the two down-stream encoded signaling proteins (*VvD1* and *VvD2*) is shown in (b). Uncropped hybridization signals and methylene blue stained lanes showing 23S (2.9 kb) and 16S (1.5 kb) bands are presented in (c). Transcription of *vvS* was not tested because the *vvR* and vvS genes overlap. The absence of a hybridization signal corresponding to a transcript covering the entire *Vv rsbRSTXD1D2* operon suggests fast turnover of the mRNA.

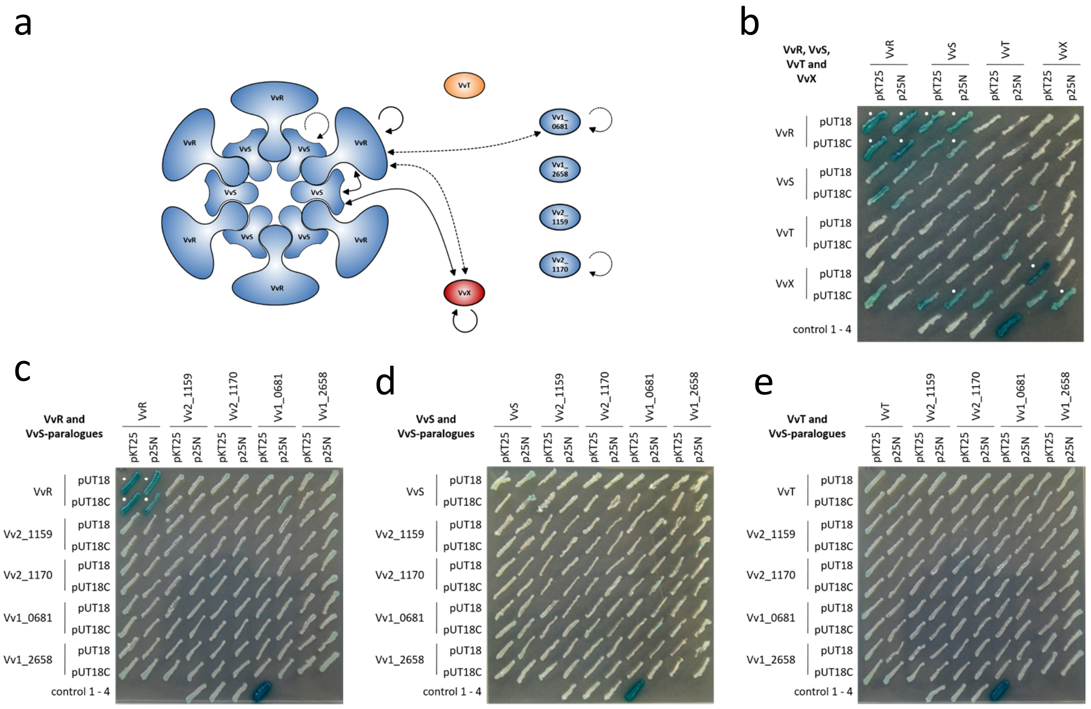

Supplementary figure 3. **BACTH analyses of *V. vulnificus* stressosome proteins.** (a) Solid lines indicate protein-protein interactions between stressosome proteins as suggested by the bacterial two-hybrid analysis. Dotted lines indicate interactions that were less well supported. Representative X-gal plates (three biologically independent samples) are shown for tested interactions between (b) RSTX-module proteins *Vv*RsbR, *Vv*RsbS, *Vv*RsbT and *Vv*RsbX as well as putative STAS domain proteins encoded outside of the RSTX-module with (c) *Vv*RsbR, (d) *Vv*RsbS and (e) *Vv*RsbT. Protein-protein interactions detected in all three replicates are highlighted by white dots.

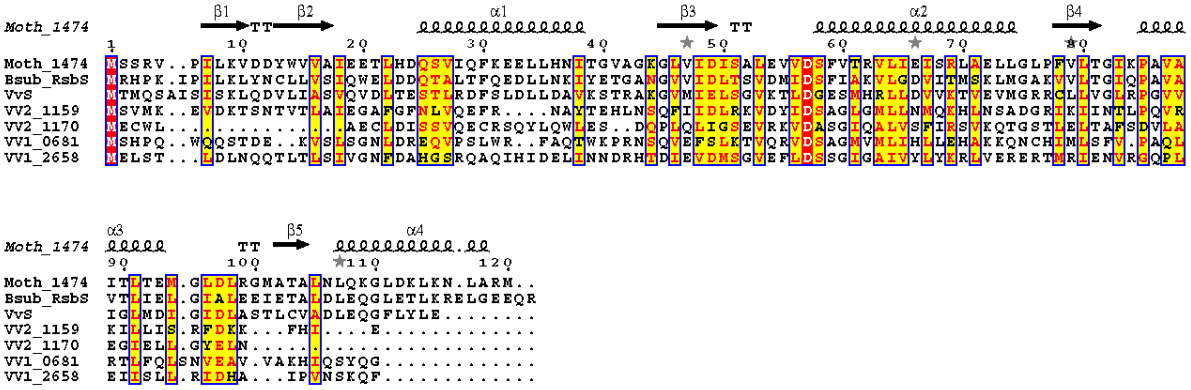

Supplementary figure 4**. Sequence alignment of *V. vulnificus* single domain STAS proteins.** *B. subtilis* and *M. thermoacetica* RsbS sequences were included to highlight the position of the regulatory serine at the N-terminus of the helix $\alpha 2$ and secondary structure elements of RsbS (*M. thermoacetica* PDB:3ZXN)[17]. Equivalent residues (70% cutoff) are indicated by yellow boxes.

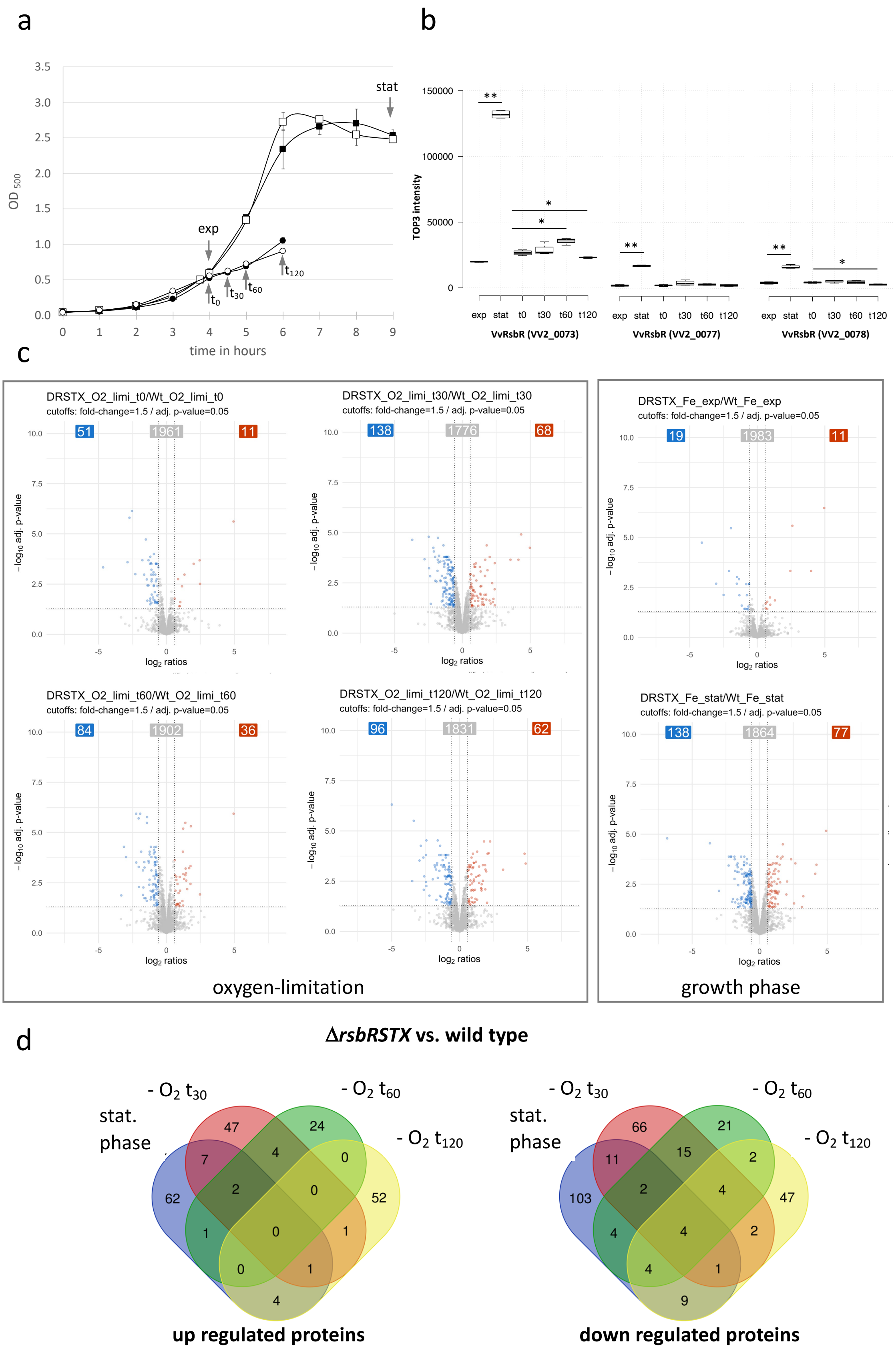
a
b
c
d
OD 500
time in hours
exp
stat
t0
t30
t60
t120
TOP3 intensity
VvRsbR (VV2_0073)
VvRsbR (VV2_0077)
VvRsbR (VV2_0078)
DRSTX_O2_limi_t0/Wt_O2_limi_t0
DRSTX_O2_limi_t30/Wt_O2_limi_t30
DRSTX_O2_limi_t60/Wt_O2_limi_t60
DRSTX_O2_limi_t120/Wt_O2_limi_t120
DRSTX_Fe_exp/Wt_Fe_exp
DRSTX_Fe_stat/Wt_Fe_stat
cutoffs: fold-change=1.5 / adj. p-value=0.05
−log10 adj. p-value
log2 ratios
oxygen-limitation
growth phase
ΔrsbRSTX vs. wild type
- O2 t30
- O2 t60
- O2 t120
stat. phase
up regulated proteins
down regulated proteins

Supplementary figure 5. **Overview of proteomic wild type *versus ΔrsbRSTX* comparison.** Growth curves of wild type (closed symbols) and *ΔrsbRSTX* mutant (open symbols) cultured in iron-supplemented (squares) and oxygen-restricted (circles) conditions. Sampling points for the proteomic analyses are indicated by arrows and error bars shown are standard deviations (a). TOP3 values of protein abundance as determined by mass spectrometry for *Vv*RsbR and the two putative downstream signaling proteins, *Vv*D1 (VV2_0077) and *Vv*D2 (VV2_0078), are shown as box plot in (b). Box-whisker extend to data points that are less than 1.5 x interquartile ranges away from 1st/3rd quartile (Tukey). Significant differences in protein accumulation are indicated by asterisk: * $p<0.05$, ** $p<0.001$. Source data for b) is available as supplementary data 1. Volcano plots showing results of proteomic wild type *versus ΔrsbRSTX* comparisons and numbers of regulated proteins according to the significance criteria: 1.5-fold change in abundance and FDR corrected $p < 0.05$ are presented in (c). Grey = total number of quantified proteins, Blue = proteins with decreased and red = increased abundance in the *ΔrsbRSTX* strain. Overlaps of regulated proteins for stationary phase and hypoxic conditions at 30 min, 60 min and 120 min are presented in (d). Proteins relating to the intersections are summarized in supplementary data 3 and 4. All source data from the proteomic analysis was deposited at the MASSive repository with accession: MSV000087636.

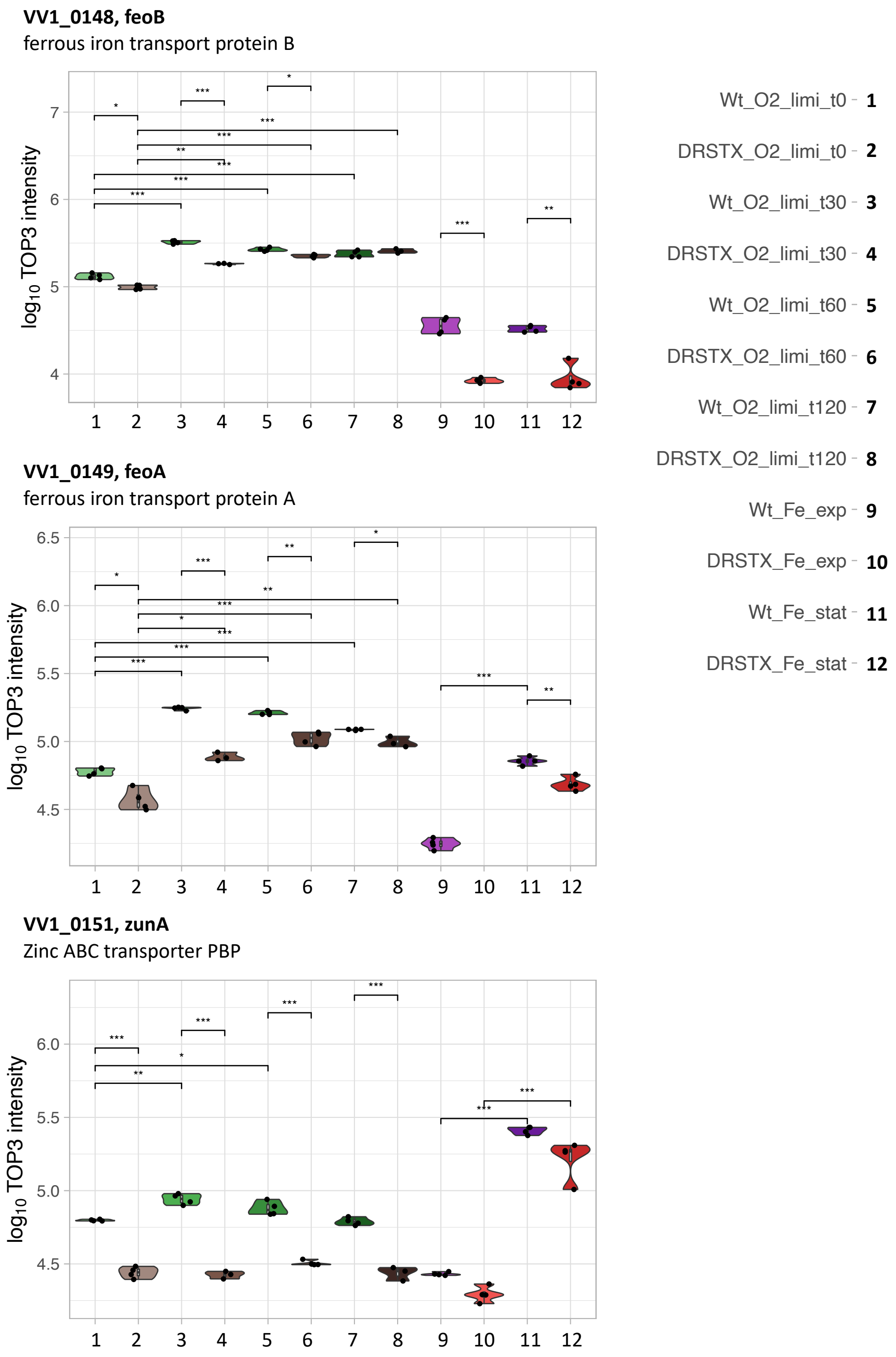

Supplementary figure 6. **Stressosome modulated changes in iron metabolism.** Violin plots showing the abundance and distribution of the individual data sets for proteins with a role in iron metabolism, which were affected by the Δ*rsbRSTX* deletion. Significant differences are labeled by asterisk: * $p<0.05$, ** $p<0.001$, *** $p<0.0001$. The legend indicating growth conditions for the individual samples is shown on the right. All source data from the proteomic analysis was deposited at the MASSive repository with accession: MSV000087636.

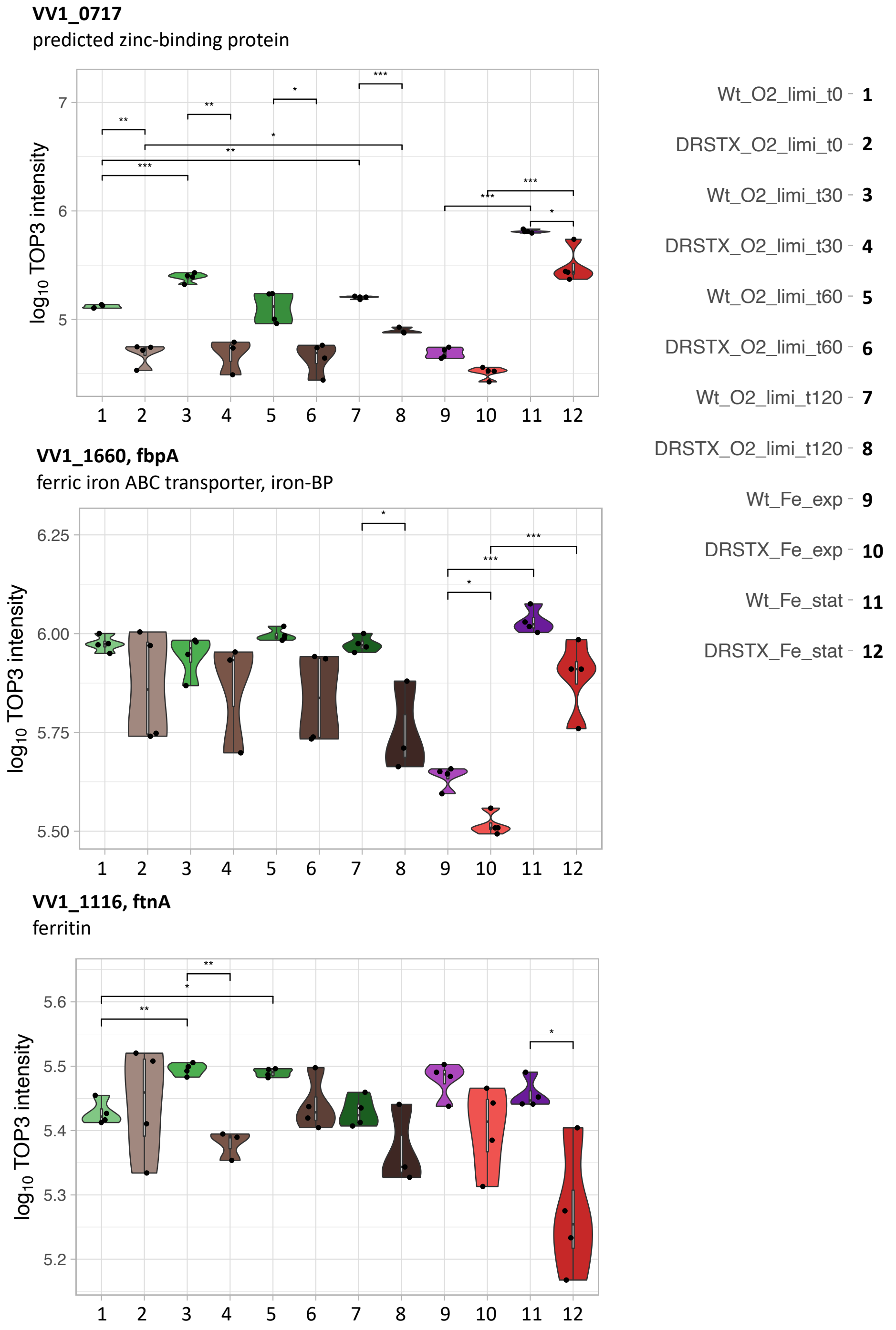

Supplementary figure 7. **Stressosome modulated changes in iron metabolism.** Violin plots showing the abundance and distribution of the individual data sets for proteins with a role in iron metabolism, which were affected by the Δ*rsbRSTX* deletion. Significant differences are labeled by asterisk: * $p<0.05$, ** $p<0.001$, *** $p<0.0001$. The legend indicating growth conditions for the individual samples is shown below the left lower graph. All source data from the proteomic analysis was deposited at the MASSive repository with accession: MSV000087636.

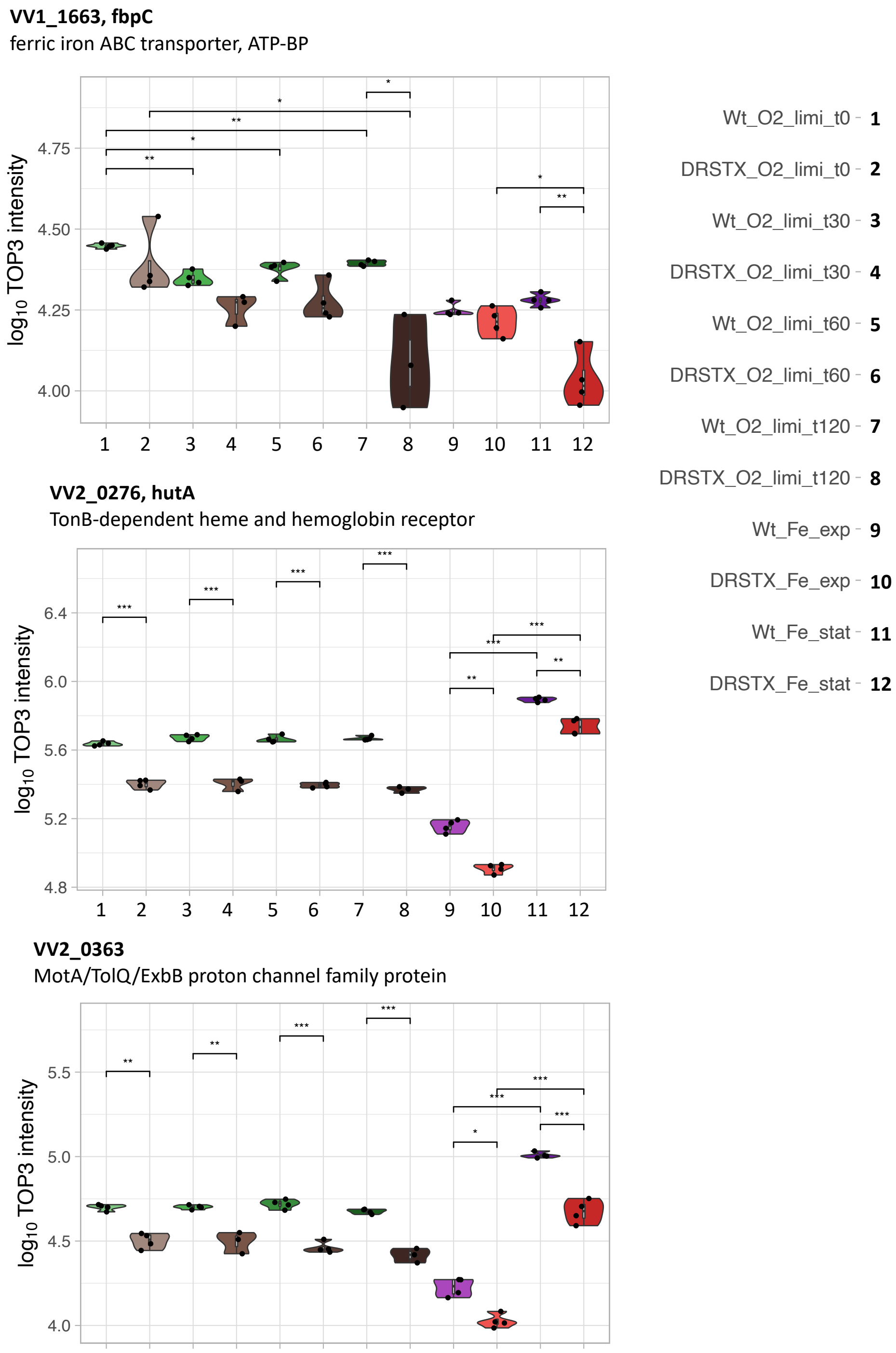

Supplementary figure 8. **Stressosome modulated changes in iron metabolism.** Violin plots showing the abundance and distribution of the individual data sets for proteins with a role in iron metabolism, which were affected by the Δ*rsbRSTX* deletion. Significant differences are labeled by asterisk: * $p<0.05$, ** $p<0.001$, *** $p<0.0001$. The legend indicating growth conditions for the individual samples is shown below the left lower graph. All source data from the proteomic analysis was deposited at the MASSive repository with accession: MSV000087636.

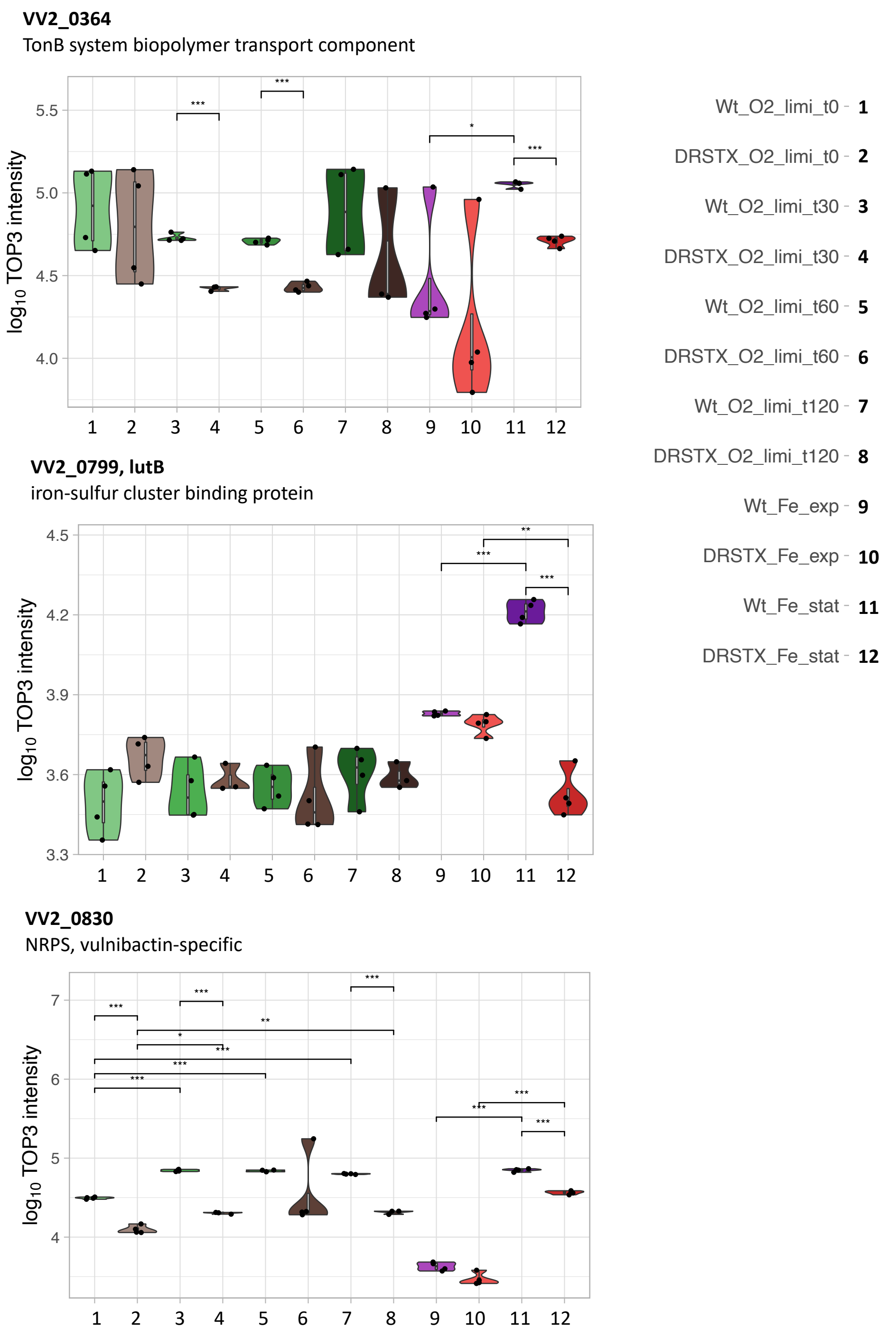

Supplementary figure 9. **Stressosome modulated changes in iron metabolism.** Violin plots showing the abundance and distribution of the individual data sets for proteins with a role in iron metabolism, which were affected by the Δ*rsbRSTX* deletion. Significant differences are labeled by asterisk: * p<0.05, ** p<0.001, *** p<0.0001. The legend indicating growth conditions for the individual samples is shown below the left lower graph. All source data from the proteomic analysis was deposited at the MASSive repository with accession: MSV000087636.

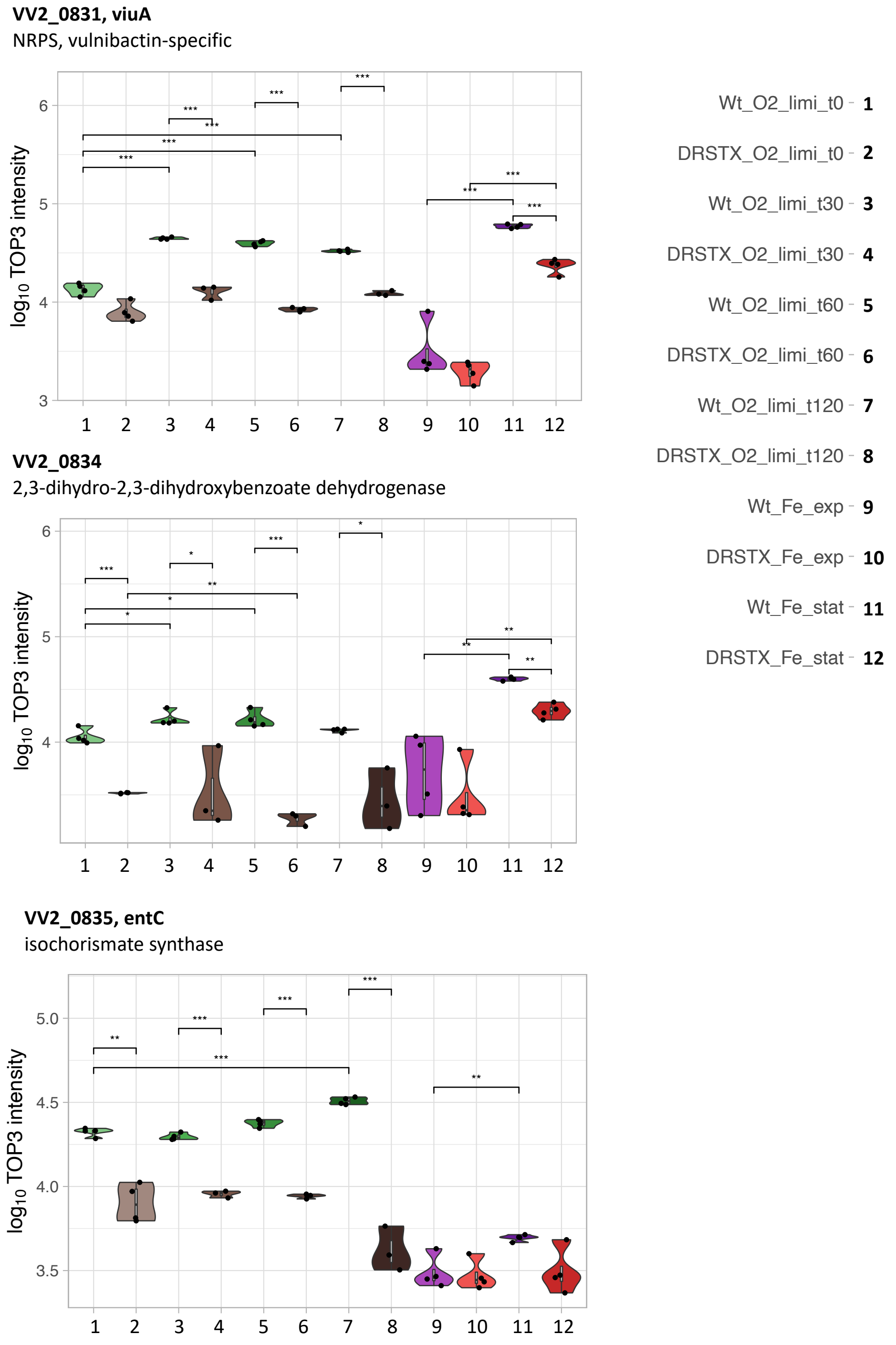

Supplementary figure 10. **Stressosome modulated changes in iron metabolism.** Violin plots showing the abundance and distribution of the individual data sets for proteins with a role in iron metabolism, which were affected by the Δ*rsbRSTX* deletion. Significant differences are labeled by asterisk: * p<0.05, ** p<0.001, *** p<0.0001. The legend indicating growth conditions for the individual samples is shown below the left lower graph. All source data from the proteomic analysis was deposited at the MASSive repository with accession: MSV000087636.

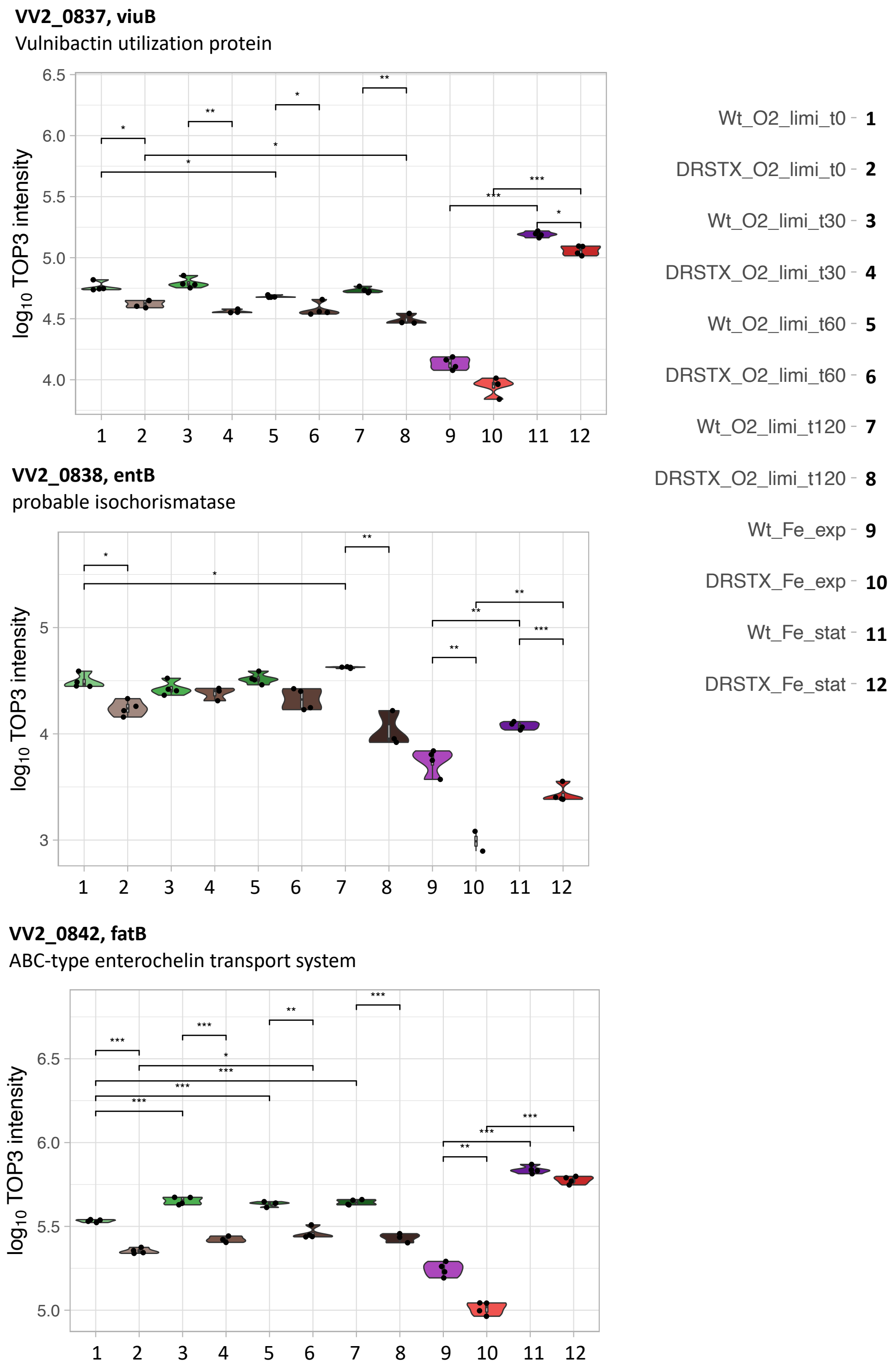

Supplementary figure 11. **Stressosome modulated changes in iron metabolism.** Violin plots showing the abundance and distribution of the individual data sets for proteins with a role in iron metabolism, which were affected by the Δ*rsbRSTX* deletion. Significant differences are labeled by asterisk: * $p<0.05$, ** $p<0.001$, *** $p<0.0001$. The legend indicating growth conditions for the individual samples is shown below the left lower graph. All source data from the proteomic analysis was deposited at the MASSive repository with accession: MSV000087636.

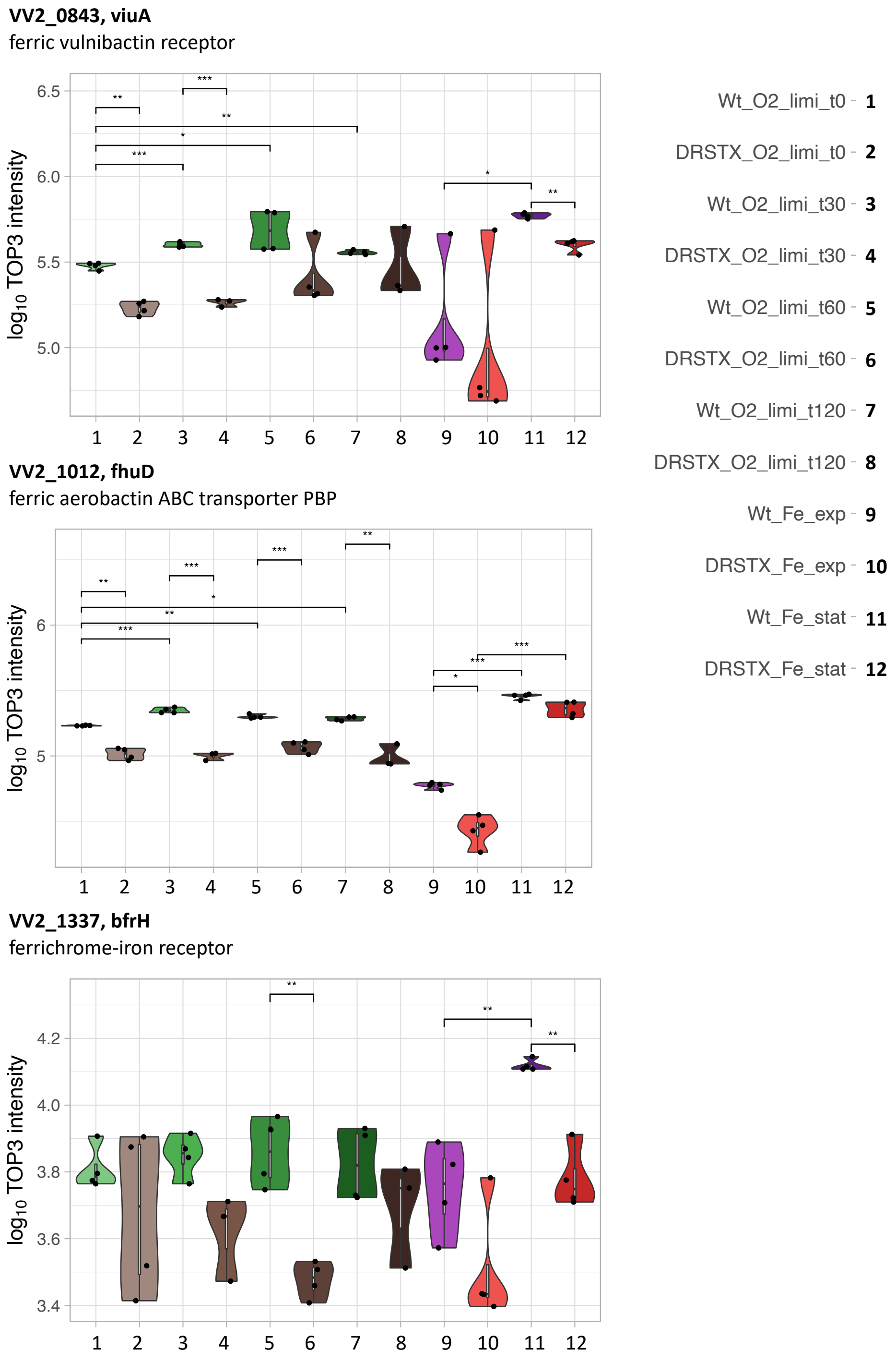

Supplementary figure 12. **Stressosome modulated changes in iron metabolism.** Violin plots showing the abundance and distribution of the individual data sets for proteins with a role in iron metabolism, which were affected by the Δ*rsbRSTX* deletion. Significant differences are labeled by asterisk: * $p<0.05$, ** $p<0.001$, *** $p<0.0001$. The legend indicating growth conditions for the individual samples is shown below the left lower graph. All source data from the proteomic analysis was deposited at the MASSive repository with accession: MSV000087636.

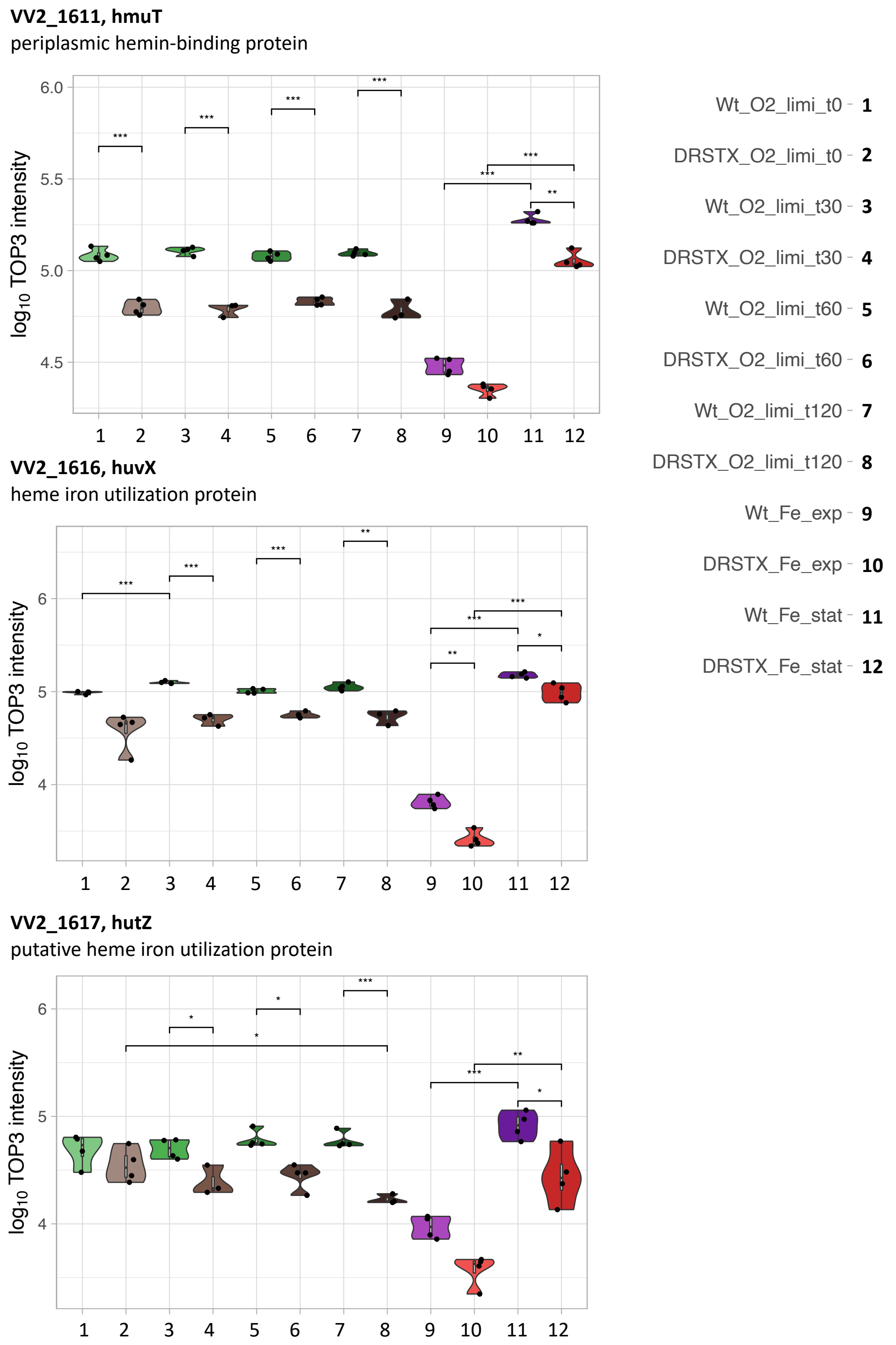

Supplementary figure 13. **Stressosome modulated changes in iron metabolism.** Violin plots showing the abundance and distribution of the individual data sets for proteins with a role in iron metabolism, which were affected by the Δ*rsbRSTX* deletion. Significant differences are labeled by asterisk: * $p<0.05$, ** $p<0.001$, *** $p<0.0001$. The legend indicating growth conditions for the individual samples is shown below the left lower graph. All source data from the proteomic analysis was deposited at the MASSive repository with accession: MSV000087636.

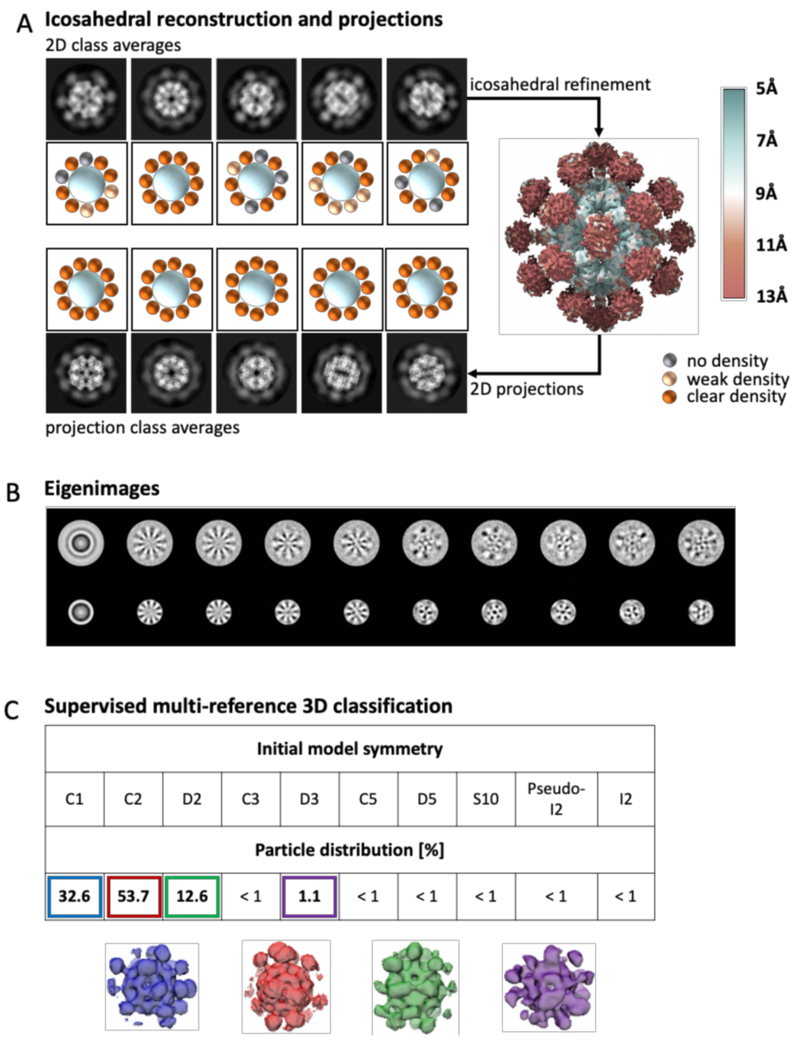

Supplementary figure 14. **Elucidating the symmetry of the *Vv*RsbRS stressosome complex.** (a) Icosahedral symmetry: Selected 2D class averages of the complex, exhibiting varying numbers of *Vv*RsbR sensory domains, are compared to 2D class averages created from random projections of an icosahedral reconstruction of the *Vv*RsbR:*Vv*RsbS complex. A clear mismatch in the number of *Vv*RsbR sensory domains is obvious for most views, proving the absence of true icosahedral symmetry in the complex. (b) Eigenvector analysis: Eigenimages created for a subset of 10,000 particles. The first ten Eigenimages exhibit clear features of 2-, 3- and 5-fold symmetry. The *Vv*RsbR:*Vv*RsbS complex core (bottom row) features more pronounced symmetry elements than the whole complex (top row). (c) Multi-reference 3D classification: The particles are classified simultaneously against a selection of ten initial models exhibiting different 2-, 3- and 5-fold symmetries. The distribution of particles in the respective 3D classes (in %) clearly shows the absence of highly symmetric complexes, and points to a predominant 2-fold symmetry in the dataset.

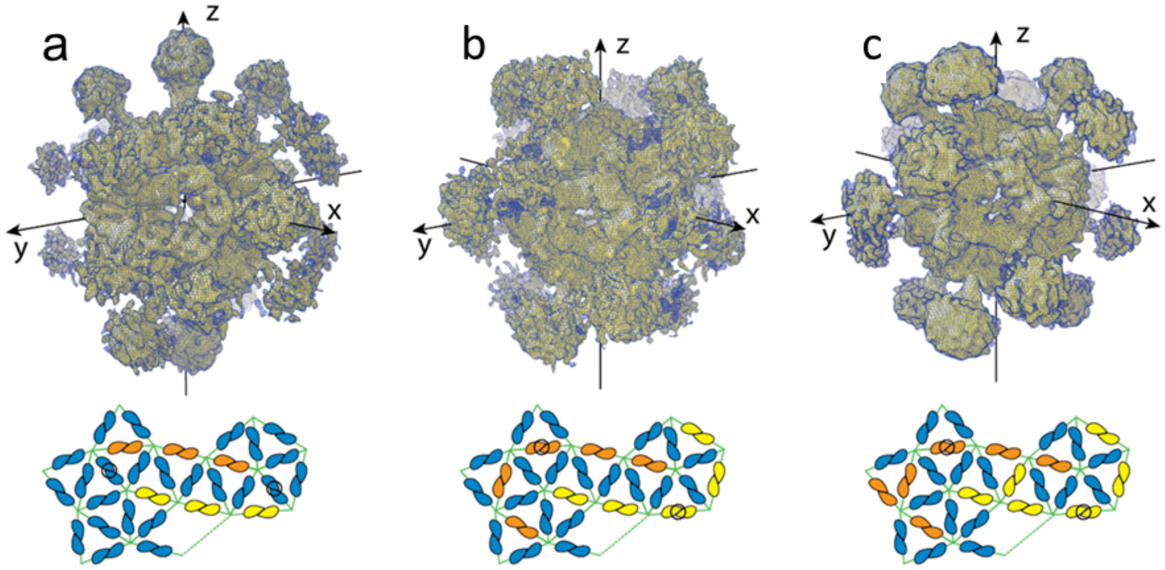

Supplementary figure 15. **The effect of different orientations of the D2 axes during reconstruction on the stoichiometry of the resulting volumes.** (a) Z- and X-axes pass through *Vv*RsbR turrets, the initial model was generated from the microscopy data. This reconstruction resulted in a 24:6 *Vv*RsbR$_2$:*Vv*RsbS$_2$ stoichiometry. (b) Y-axis passes through turrets, EMDB1555 (*B. subtilis*) was used as an initial model. The resulting *Vv*RsbR$_2$:*Vv*RsbS$_2$ stoichiometry was 20:10, as described for the *B. subtilis* stressosome. (c) Y-axis passes through turrets but X- and Z-axes pass through *Vv*RsbS. As the initial model, the reconstruction volume shown in B was used. In this reconstruction, the stoichiometry was 18:12 *Vv*RsbR$_2$:*Vv*RsbS$_2$. In all reconstructed volumes, off-axis dimers are different. Unfolded views of the complex arrangement are depicted below the respective volume, with *Vv*RsbS shown in yellow and orange, and *Vv*RsbR shown in blue. The positions where the Z-axis passes the reconstructed volumes are depicted as circles on the unfolded views. The differences in *Vv*RsbR:*Vv*RsbS complex stoichiometry, resulting from the different orientations of the D2 axes during reconstruction of the volumes, can be captured easily from the unfolded views.

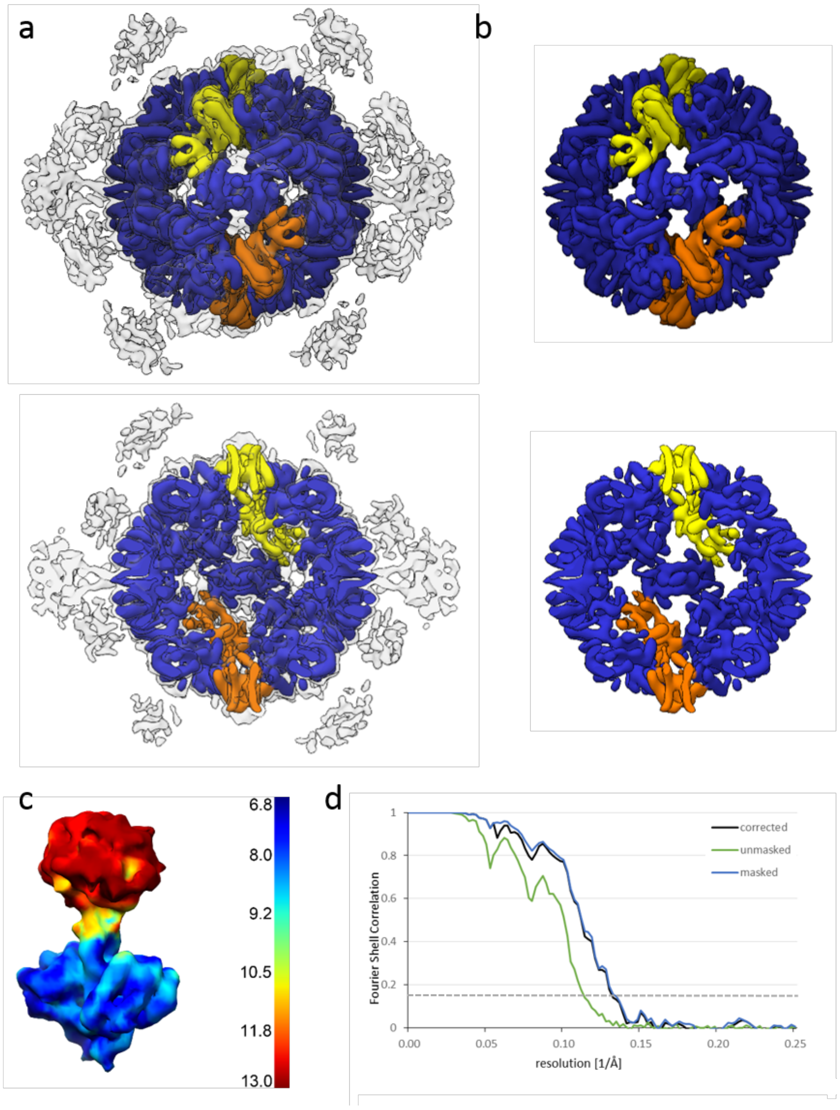

Supplementary figure 16. **cryo-EM density map of the *Vv*RsbRS complex.** (a, b) The D2-symmetric cryo-EM density map of the whole complex (grey, transparent surface) is shown at a threshold of 0.06, overlaid with the density map of the STAS domain core (colored surface), rendered at a threshold of 0.15. A complete (top) and cut-open (bottom) view are shown, respectively. *Vv*RsbR dimers are depicted in blue, *Vv*RsbS dimers in yellow and orange. (c) The local resolution distribution is illustrated for one *Vv*RsbR dimer; the resolution in the STAS domain extends well below 7Å, while the sensory domains are less well resolved. (d) The FSC curve for the *Vv*RsbRS complex core (b) indicates a global resolution of 7.8Å according to the FSC = 0.143 criterion (dashed grey line). Due to considerable variations in the densities of the *Vv*RsbR sensory domains (a), the turrets have been masked out to obtain a more focused volume reconstruction for the STAS domain core.

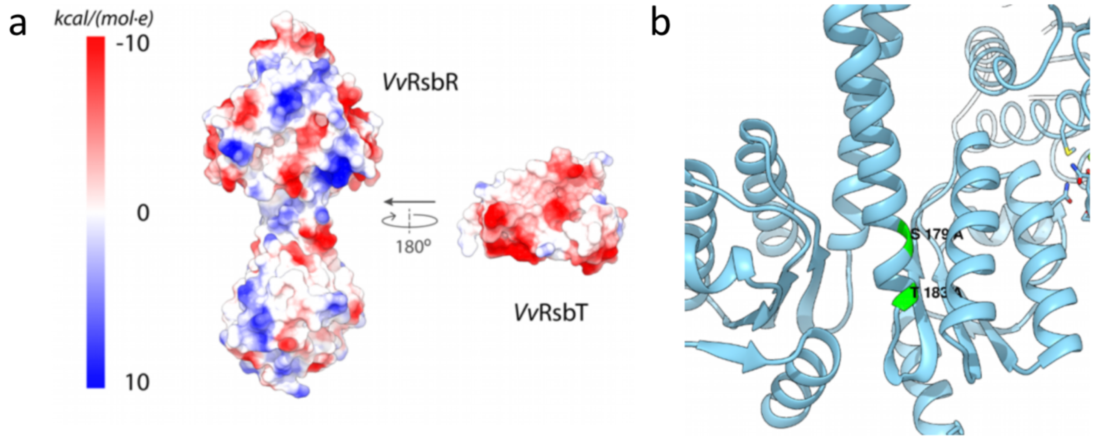

Supplementary figure 17. **Phosphorylation and coulombic surface of the *Vv*RsbR:*Vv*RsbT complex.** (A) The two residues found to be phosphorylated *in vitro*, Ser178 and Thr182, of the *V. brasiliensis Vb*RsbR[18] are buried in the linker-STAS domain interface in *V. vulnificus Vv*RsbR. (B) Complementarity of charges is only weakly represented in *Vv*RsbR and *Vv*RsbT (compare to (back-to-back submission Miksys *et al.*).

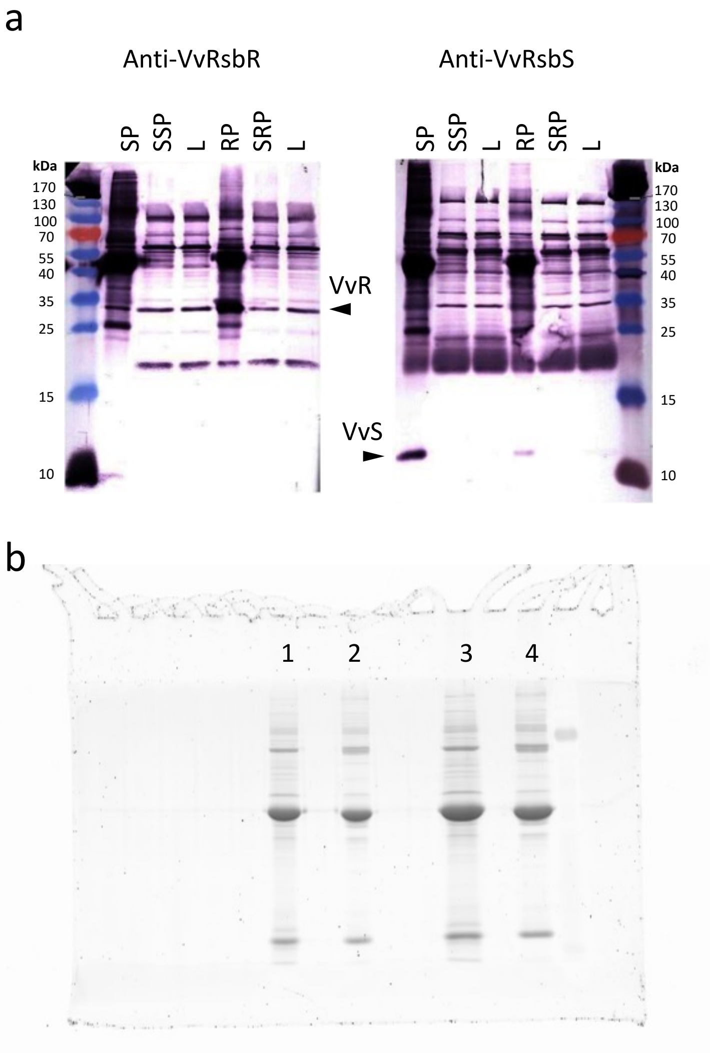

Supplementary figure 18**. Immune precipitation of *Vv*RsbR:*Vv*RsbS complex from *V. vulnificus* cell extracts and recombinant *Vv*RsbR:*Vv*RsbS complex produced in *E. coli***For the *in vivo* detection of *Vv*RsbR:*Vv*RsbS complexes, the soluble protein fraction from stationary phase *V. vulnificus* cells grown in iron supplemented minimal medium was subjected to immune precipitation using either *Vv*RsbR or *Vv*RsbS specific polyclonal sera. The uncropped blot is shown in a). Precipitates and control samples were blotted onto membranes, which were then incubated with either anti-*Vv*RsbR or anti-*Vv*RsbS as primary antibodies to analyze co-precipitation of *Vv*RsbR:*Vv*RsbS. Bound *Vv*RsbR and *Vv*RsbS specific antibodies (arrows) were detected with anti-rabbit IgG. In addition to anti-*Vv*RsbR and anti-*Vv*RsbS, the secondary anti-rabbit IgG also detected a band around 55 kDa (asterisk) in the precipitation reaction, which corresponds to the heavy chain of the anti-*Vv*RsbR antibody used for precipitation. The anti-

*Vv*RsbS antibody did not precipitate recognizable amounts of *Vv*RsbR, while *Vv*RsbS was clearly present in the anti-*Vv*RsbR precipitate. This may result from interference of anti-*Vv*RsbS binding to *Vv*RsbS with *Vv*RsbR:*Vv*RsbS complex formation. L: lysate used for immune precipitation, SP: *Vv*RsbS precipitate, RP: *Vv*RsbR precipitate, SSP: supernatant of *Vv*RsbS precipitation, SSR: supernatant of *Vv*RsbR precipitation. b) Uncropped gel of purified *Vv*RsbR:*Vv*RsbS stressosome complex (lane 1-4) separated by SDS PAGE and stained with Krypton (Thermo Scientific). The gel was imaged with a Typhoon fluorescence scanner, with excitation at 532 nm and emission recorded at 560 nm. Bands corresponding to *Vv*RsbR and *Vv*RsbS are indicated. Lane 4 corresponds to the band shown in figure 2d.

SUPPLEMENTARY TABLES

Supplementary table 1 primers used in this study		
name	Primer sequence (5´-3´)[1]	purpose
**Protein expression**		
4	ATGGTACGTCTCAAATGTCACTGGGAAGTGTATTAAATAAAGT	VvR-for,VvRS co-expression
16	**ATCGGCTTCTCCACCTTTTG**CTACTCATGAGGAAAGTGT	VvR-for,VvRS co-expression
15	TAG**CAAAAGGTGGAGAAGCCGAT**ATGACGATGCAAAGTGCAA	VvR-for,VvRS co-expression
5	ATGGTACGTCTCAGCGCTTTATTCCAAATAAAGAAACCCCTGCTC	VvR-for,VvRS co-expression
VV2_0073-	ATGGTAGGTCTCAAATGTCACTGGGAAGTGTATTAAATAAAGT	for VvR165 N-term
VV2_0073-	ATGGTAGGTCTCAGCGCTCTATTTATTAATTAAACGCGAATAGGTATC	rev VvR165 N-term
VV2_0073-	ATGGTAGAAGACAAAATGTCACTGGGAAGTGTATTAAATAAAGT	VvR-for, VvR-strep
VV2_0073-	ATGGTAGAAGACAAGCGCTCTCATGAGGAAAGTGTTTCAAACC	VvR-rev, VvR-strep
VV2_0074-	ATGGTAGGTCTCAAATGACGATGCAAAGTGCAATTTCGATTT	VvS-for, VvS-strep
VV2_0074-	ATGGTAGGTCTCAGCGCTTTCCAAATAAAGAAACCCCTGCTC	VvS-rev, VvS-strep
77	TAATACGACTCACTATAGGG	pPR-IBA1-for, sequencing
78	TAGTTATTGCTCAGCGGTGG	pPR-IBA1-rev, sequencing
**Northern blot analyses**		
329	ATGTCACTGGGAAGTGTATT	*vvR*-for, probe
508	CTAATACGACTCACTATAGGGAGCGATAGAAGGCGAAACGCCC	*vvR-rev,* probe
511	ATGGTAGAAGTGGTCAGAAGCC	*vvT*-for, probe
512	CTAATACGACTCACTATAGGGAGTCACACCCACTTCTCAGC	*vvT-rev,* probe
513	ATGGGATTTGACTTTGTTAC	*vvX*-for, probe
514	CTAATACGACTCACTATAGGGAGTCAGTCATAGTAGTACCTAACTGC	*vvX-rev,* probe
331	ATGACTGAATCGATCTCTTTAACC	*vvD1*-for, probe
332	CTAATACGACTCACTATAGGGAGTGGAGACCCCCAAATCGATC	*vvD1-rev,* probe
333	ATGGAAGGGAATCGTGACACTG	*vvD2*-for, probe
334	CTAATACGACTCACTATAGGGAGGATAGCCAGAACCATCCCAC	*vvD2*-rev, probe
**BACTH analyses**		
549	GCTCTAGAGTCACTGGGAAGTGTATTAAATAAAG	VV2_0073-for
550	GCGGTACCCGCTCATGAGGAAAGTGTTTCAAACC	VV2_0073-rev
551	GCTCTAGAGACGATGCAAAGTGCAATTTCG	VV2_0074-for
552	GCGGTACCCGTTCCAAATAAAGAAACCCCTG	VV2_0074-rev
553	GCTCTAGAGGTAGAAGTGGTCAGAAGCCTAC	VV2_0075-for
554	GCGGTACCCGCACCCACTTCTCAGCAACAATC	VV2_0075-rev
555	GCTCTAGAGGGATTTGACTTTGTTACTGTC	VV2_0076-for
556	GCGGTACCCGGTCATAGTAGTACCTAACTGC	VV2_0076-rev
557	GCGGATCCCACTGAATCGATCTCTTTAACC	VV2_0077-for
558	GCGGTACCCGCGATTCCCTTCCATTTTTCTC	VV2_0077-rev
559	GCTCTAGAGGAAGGGAATCGTGACACTGTG	VV2_0078-for
560	GCGGTACCCGTCTGGCACTGGTTTTCTGGG	VV2_0078-rev
561	GCTCTAGAGTCAGTGATGAAAGAAGTCGATAAAAC	VV2_1159-for
562	GCGGTACCCGCTCGATGTGGAATTTTTTATCGAAAC	VV2_1159-rev
563	GCTCTAGAGGAATGTTGGCTGGCTGAGTG	VV2_1170-for
564	GCGGTACCCGATTAAGCTCATAACCCAGC	VV2_1170-rev
565	GCTCTAGAGTCACACCCGCAATGGCAGCAATC	VV1_0681-for
566	GCGGTACCCGCCCCTGATAACTTTGAATATG	VV1_0681-rev
567	GCTCTAGAGGAACTCAGCACACTCGACCTC	VV1_2658-for
568	GCGGTACCCGAAACTGCTTGGAGTTAACGG	VV1_2658-rev
391	CACTTTATGCTTCCGGCTCG	pUT18-for and p25-N-for,
392	GGAACGGGCGCCGGCGCGAGC	pUT18-rev, sequencing
393	CGAGCGGACGTTCGAAGTTCTC	pUT18C-for, sequencing
394	GTTGGCGGGTGTCGGGGCTG	pUT18C-rev, sequencing
388	CGGCGGATATCGACATGTTCGCC	pKT25-for, sequencing
389	GCTGGCGAAAGGGGGATGTGCTGC	pKT25-rev, sequencing
390	CCTTGATGCCATCGAGTACGGC	p25-N-rev, sequencing

1) The sequence, corresponding to the 20 nucleotides upstream of the VV2_0073 gene, introduced between VvR (VV2_0073) and VvS (VV2_0074) to generate an additional ribosomal binding site.

SUPPLEMENTARY REFERENCES

1. Yang, X., Kang, C. M., Brody, M. S. & Price, C. W. Opposing pairs of serine protein kinases and phosphatases transmit signals of environmental stress to activate a bacterial transcription factor. *Genes Dev.* **10**, 2265–2275 (1996).

2. Gaidenko, T. A., Yang, X., Lee, Y. M. & Price, C. W. Threonine phosphorylation of modulator protein RsbR governs its ability to regulate a serine kinase in the environmental stress signaling pathway of Bacillus subtilis. *J. Mol. Biol.* **288**, 29–39 (1999).
3. Kang, C. M., Vijay, K. & Price, C. W. Serine kinase activity of a Bacillus subtilis switch protein is required to transduce environmental stress signals but not to activate its target PP2C phosphatase. *Mol. Microbiol.* **30**, 189–196 (1998).
4. Hardwick, S. W. *et al.* Structural and functional characterization of partner switching regulating the environmental stress response in Bacillus subtilis. *J. Biol. Chem.* **282**, 11562–72 (2007).
5. Delumeau, O. *et al.* Functional and structural characterization of RsbU, a stress signaling protein phosphatase 2C. *J. Biol. Chem.* **279**, 40927–40937 (2004).
6. Benson, A. K. & Haldenwang, W. G. Bacillus subtilis sigma B is regulated by a binding protein (RsbW) that blocks its association with core RNA polymerase. *Proc. Natl. Acad. Sci. U. S. A.* **90**, 2330–4 (1993).
7. Alper, S., Dufour, A., Garsin, D. A., Duncan, L. & Losick, R. Role of adenosine nucleotides in the regulation of a stress-response transcription factor in Bacillus subtilis. *J. Mol. Biol.* **260**, 165–77 (1996).
8. Marles-Wright, J. *et al.* Molecular architecture of the 'stressosome,' a signal integration and transduction hub. *Science* **322**, 92–6 (2008).
9. Chen, C. C., Lewis, R. J., Harris, R., Yudkin, M. D. & Delumeau, O. A supramolecular complex in the environmental stress signalling pathway of Bacillus subtilis. *Mol. Microbiol.* **49**, 1657–1669 (2003).
10. Kim, T. J., Gaidenko, T. A. & Price, C. W. A multicomponent protein complex mediates environmental stress signaling in Bacillus subtilis. *J. Mol. Biol.* **341**, 135–150 (2004).
11. Kim, T. J., Gaidenko, T. A. & Price, C. W. In vivo phosphorylation of partner switching regulators correlates with stress transmission in the environmental signaling pathway of Bacillus subtilis. *J. Bacteriol.* **186**, 6124–6132 (2004).

12. Eymann, C. *et al.* In vivo phosphorylation patterns of key stressosome proteins define a second feedback loop that limits activation of Bacillus subtilis σB. *Mol. Microbiol.* **80**, 798–810 (2011).

13. Chen, C. C., Yudkin, M. D. & Delumeau, O. Phosphorylation and RsbX-dependent dephosphorylation of RsbR in the RsbR-RsbS complex of Bacillus subtilis. *J. Bacteriol.* **186**, 6830–6836 (2004).

14. Vijay, K., Brody, M. S., Fredlund, E. & Price, C. W. A PP2C phosphatase containing a PAS domain is required to convey signals of energy stress to the sigmaB transcription factor of Bacillus subtilis. *Mol. Microbiol.* **35**, 180–8 (2000).

15. Brody, M. S., Vijay, K. & Price, C. W. Catalytic function of an alpha/beta hydrolase is required for energy stress activation of the sigma(B) transcription factor in Bacillus subtilis. *J. Bacteriol.* **183**, 6422–8 (2001).

16. Murray, J. W., Delumeau, O. & Lewis, R. J. Structure of a nonheme globin in environmental stress signaling. *Proc. Natl. Acad. Sci. U. S. A.* **102**, 17320–17325 (2005).

17. Quin, M. B. *et al.* The bacterial stressosome: A modular system that has been adapted to control secondary messenger signaling. *Structure* **20**, 350–363 (2012).

18. Jia, X., Wang, J., Rivera, S., Duong, D. & Weinert, E. E. An O2-sensing stressosome from a Gram-negative bacterium. *Nat. Commun.* **7**, 12381 (2016).
