# Supplementary material for: The Vibrio vulnificus stressosome is an oxygen-sensor involved in regulating iron metabolism
Source: Commun Biol. 2022 Jun 27;5:622. doi: 10.1038/s42003-022-03548-w (PMC9237108; doi:10.1038/s42003-022-03548-w)
Supplement: Supplementary file 3 — Description of Additional Supplementary Files [file 42003_2022_3548_MOESM3_ESM.pdf]

Supplementary Data 1:
Source data for Fig 1

Supplementary Data 2:
Source data for Supp Fig 5

Supplementary Data 3:
Results Venn diagram analysis of proteins up-regulated in ΔrsbRSTX versus wild type comparison. (relevant to Fig 5)

Supplementary Data 4:
Results Venn diagram analysis of proteins down-regulated in ΔrsbRSTX versus wild type comparison. (relevant to Fig 5)

Supplementary Data 5:
The occurrence of stressosome gene clusters in the genus Vibrio
