# Supplementary material for: The Vibrio vulnificus stressosome is an oxygen-sensor involved in regulating iron metabolism
Source: Commun Biol. 2022 Jun 27;5:622. doi: 10.1038/s42003-022-03548-w (PMC9237108; doi:10.1038/s42003-022-03548-w)
Supplement: Supplementary file 9 — Reporting summary [file 42003_2022_3548_MOESM9_ESM.pdf]

nature research

Corresponding author(s): Jan Pané-Farré
Christine Ziegler

Last updated by author(s): May 18, 2022

Reporting Summary

Nature Research wishes to improve the reproducibility of the work that we publish. This form provides structure for consistency and transparency in reporting. For further information on Nature Research policies, see our Editorial Policies and the Editorial Policy Checklist.

Statistics

For all statistical analyses, confirm that the following items are present in the figure legend, table legend, main text, or Methods section.

n/a	Confirmed	
☐	☒	The exact sample size (*n*) for each experimental group/condition, given as a discrete number and unit of measurement
☐	☒	A statement on whether measurements were taken from distinct samples or whether the same sample was measured repeatedly
☐	☒	The statistical test(s) used AND whether they are one- or two-sided<br>*Only common tests should be described solely by name; describe more complex techniques in the Methods section.*
☒	☐	A description of all covariates tested
☐	☒	A description of any assumptions or corrections, such as tests of normality and adjustment for multiple comparisons
☐	☒	A full description of the statistical parameters including central tendency (e.g. means) or other basic estimates (e.g. regression coefficient) AND variation (e.g. standard deviation) or associated estimates of uncertainty (e.g. confidence intervals)
☐	☒	For null hypothesis testing, the test statistic (e.g. *F*, *t*, *r*) with confidence intervals, effect sizes, degrees of freedom and *P* value noted<br>*Give P values as exact values whenever suitable.*
☐	☒	For Bayesian analysis, information on the choice of priors and Markov chain Monte Carlo settings
☒	☐	For hierarchical and complex designs, identification of the appropriate level for tests and full reporting of outcomes
☒	☐	Estimates of effect sizes (e.g. Cohen's *d*, Pearson's *r*), indicating how they were calculated

Our web collection on statistics for biologists contains articles on many of the points above.

Software and code

Policy information about availability of computer code

Data collection	CryoEM: Cryo-EM data collection was performed manually over multiple sessions on a Tecnai Polara (Thermo Fisher Scientific) cryo-electron microscope (operated at 300 keV), equipped with a Falcon II detector (Thermo Fisher Scientific).<br>Proteomics: MassLynx™ Software Version V4.1 (Waters Corporation)
Data analysis	CryoEM:RELION, RELION-2, MotionCor, CTFFIND4.1, SPIDER - PCA<br>Structure homology modeling: HHblits, PSIPRED, CHIMERA, COOT, FLEX-EM, MODELLER<br>Proteomics: PLGS v3.3 (Water Corporation), ISOQuant 1.8,FactoMineR package (version 2.3), PECA package (version 1.24.0), R version 4.0.2 using the tidyverse package (version 1.3.0), limma package (version 3.44.3), ggplot2 package (version 3.3.2), Venn diagrams were drawn using the tool available at: http://bioinformatics.psb.ugent.be/beg/software. A modified t-statistic is calculated using the linear modeling approach in the Bioconductor limma package.

For manuscripts utilizing custom algorithms or software that are central to the research but not yet described in published literature, software must be made available to editors and reviewers. We strongly encourage code deposition in a community repository (e.g. GitHub). See the Nature Research guidelines for submitting code & software for further information.

Data

Policy information about availability of data

All manuscripts must include a data availability statement. This statement should provide the following information, where applicable:

- Accession codes, unique identifiers, or web links for publicly available datasets
- A list of figures that have associated raw data
- A description of any restrictions on data availability

Proteomic data that support the findings of this study are available at MASSive with accession: MSV000087636. The structural data presented in this manuscript has

April 2020

been deposited in the wwPDB with the ID 7O01. The corresponding EM map can be found in the EMDB under the code EMD-12676. All other data are available from the corresponding author on reasonable request.

Field-specific reporting

Please select the one below that is the best fit for your research. If you are not sure, read the appropriate sections before making your selection.

- [x] Life sciences
- [ ] Behavioural & social sciences
- [ ] Ecological, evolutionary & environmental sciences

For a reference copy of the document with all sections, see nature.com/documents/nr-reporting-summary-flat.pdf

Life sciences study design

All studies must disclose on these points even when the disclosure is negative.

Sample size	sample size 3-4 independent biological replicates
Data exclusions	no data excluded
Replication	all attempts at replication were sucessful
Randomization	samples were allocated in groups according to genotype (wild type, stressosome mutant) or cultivation procedure (with/without iron or oxygen, exponential growth and stationary phase)
Blinding	no blinding

Reporting for specific materials, systems and methods

We require information from authors about some types of materials, experimental systems and methods used in many studies. Here, indicate whether each material, system or method listed is relevant to your study. If you are not sure if a list item applies to your research, read the appropriate section before selecting a response.

Materials & experimental systems

n/a	Involved in the study	
☐	☒	Antibodies
☒	☐	Eukaryotic cell lines
☒	☐	Palaeontology and archaeology
☒	☐	Animals and other organisms
☒	☐	Human research participants
☒	☐	Clinical data
☒	☐	Dual use research of concern

Methods

n/a	Involved in the study	
☒	☐	ChIP-seq
☒	☐	Flow cytometry
☒	☐	MRI-based neuroimaging

Antibodies

Antibodies used	poly-clonal Anti VvRsbR, VvRsbS, produced in rabbits
Validation	pre-immune serum, mutant strains lacking target protein
